# Lupin protein isolate improves insulin sensitivity and steatohepatitis in vivo and modulates the expression of the *Fasn*, *Gys2*, and *Gsk3b* genes

**DOI:** 10.1002/fsn3.2206

**Published:** 2021-03-08

**Authors:** Irma Catalina Soto‐Luna, Pedro Macedonio García‐López, Belinda Vargas‐Guerrero, Tereso Jovany Guzmán, José Alfredo Domínguez‐Rosales, Carmen Magdalena Gurrola‐Díaz

**Affiliations:** ^1^ Instituto de Investigación en Enfermedades Crónico‐Degenerativas Instituto Transdisciplinar de Investigación e Innovación en Salud Departamento de Biología Molecular y Genómica Centro Universitario de Ciencias de la Salud Universidad de Guadalajara Guadalajara Mexico; ^2^ Departamento de Botánica y Zoología Centro Universitario de Ciencias Biológicas y Agropecuarias Universidad de Guadalajara Zapopan Mexico; ^3^ Department of Pharmaceutical and Medicinal Chemistry University of Münster Münster Germany

**Keywords:** carbohydrate metabolism, fatty liver, high‐fat diet, insulin resistance, lipid metabolism

## Abstract

Although studies on lupin protein isolate (LPI) have indicated the presence of a preventive effect on insulin resistance (IR) and lipid disturbances, their influence on established pathological traits has received little attention. Here, we evaluated the in vivo effects of LPI on IR and steatohepatitis as well as its influence on genes involved in lipid and carbohydrate metabolism. We first induced IR and steatohepatitis in rats by maintaining them on a high‐fat diet for 5 weeks. Thereafter, we administered LPI to the rats daily for 3 weeks. LPI improved insulin sensitivity (AUC: 6,777 ± 232 vs. 4,971 ± 379, *p* < .05, pre‐ vs. post‐treatment values) and reduced glucose and triglyceride levels by one‐third. In addition, LPI‐treated rats exhibited attenuated steatohepatitis. At the molecular level, LPI treatment reduced liver *Fasn* gene expression substantially but increased *Gys2* and *Gsk3b* levels. We concluded that the hypolipidemic and hypoglycemic activities of LPI may be caused by reduced liver lipogenesis and modulation of insulin sensitization mechanisms.

## Graphical abstract

Here, we evaluated the in vivo effects of LPI on IR and steatohepatitis, and its influence on lipid and carbohydrate metabolism genes. LPI improved insulin sentitivity and reduced glucose and triglyceride levels. Besides, LPI‐treated rats exhibited attenuated steatohepatitis. reduced liver *Fasn* gene expression substantially, but increased *Gys2* and *Gsk3b* levels. LPI hypolipidemic and hypoglycemic activity may result from reduced liver lipogenesis and the modulation of insulin sensitization‐mechanisms.

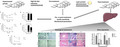

## 1 INTRODUCTION

Insulin resistance (IR) is a condition related to metabolic alterations such as hyperglycemia and hypertriglyceridemia and is the first of multiple hits that determine the progression of nonalcoholic fatty liver disease (NAFLD) to nonalcoholic steatohepatitis (NASH; Reaven, 1995). Moreover, the clinical features of NAFLD are similar to those present in metabolic disorders, such as obesity, inflammation, IR, and type 2 diabetes (T2D; Liu et al., 2016; Samuel et al., 2004). Current therapeutic strategies for IR management initially focus on encouraging lifestyle changes, including forming healthy eating habits and performing daily exercise, followed by treatment with pharmacological agents (Cornier et al., 2005; Devlin, 1992; McAuley et al., 2003). In particular, the use of legume seed proteins for the management and treatment of noncommunicable diseases (NCDs) is an area of current interest, with an increasing number of recent reports regarding this (Hosseinpour‐Niazi et al., 2015; McMacken & Shah, 2017; Pihlanto et al., 2017; Rizkalla et al., 2002).

Legumes have adequate nutritional value for a healthy diet because of their protein, fiber, complex carbohydrates, and micronutrient content (Polak et al., 2015). Besides their nutritional properties, legumes contain a wide variety of health‐promoting bioactive compounds (Rebello et al., 2014). Regarding the importance of legume seeds in diabetes management, clinical studies have supported its inclusion in the diet because of its purported insulin sensitization actions (Clark et al., 2018).

The legume lupin (genus *Lupinus*) belongs to the Fabaceae family and includes more than 300 species distributed worldwide. The domestic species *Lupinus albus*, *L*. *angustifolius*, *L*. *luteus*, and *L*. *mutabilis* are the most widely cultivated and most widely used lupins for animal and human consumption in various countries (Duranti et al., 2008; Kohajdová et al., 2011). Furthermore, lupin and soybean represent excellent protein sources for the human diet (Erbaş et al., 2005; Sirtori et al., 2004).

Studies on humans and laboratory animals have shown that adding soybean protein to the diet lowers total cholesterol, LDL‐cholesterol (LDL‐c), and triglyceride serum levels as well as ameliorates IR (Anderson et al., 1995; Tachibana et al., 2014). Moreover, soybean protein supplementation has been associated with clinical improvements in metabolic syndrome and T2D. Reduced values of fasting plasma glucose, insulin, homeostatic model assessment of IR (HOMA‐IR), total cholesterol, low‐density lipoprotein cholesterol, diastolic blood pressure, and C‐reactive protein have also been found in subjects after consuming soybean protein (Zhang et al., 2016). Similarly, other studies have shown beneficial effects of lupin protein on hyperlipidemia and hyperglycemia (Bouchoucha et al., 2016; Sewani‐Rusike et al., 2015). In rats fed with lupin protein‐supplemented pasta, body weight gain and food intake were reduced (Capraro et al., 2014). In addition, the consumption of a lupin protein‐based beverage caused acute reductions in serum glucose levels in T2D patients (Dove et al., 2011). Fornasini et al. (2012) found that lupin protein did not reduce glycemia in normoglycemic individuals but induced a hypoglycemic effect in dysglycemic individuals.

Previous research in the nutraceutical field has revealed the potential use of lupin protein in regulating IR, lipid, and glucose metabolism. One mechanism related to the metabolic effects of insulin is the modulation of liver glycogen synthesis, mainly through the activation of the glycogen synthase enzyme. The increase in liver glycogen synthesis has been associated with improved glucose tolerance (Ros et al., 2010). Specifically, *Gys2* and *Gsk3b* are directly involved in the glycogen synthesis pathway. In contrast, altered lipid metabolism has also been implicated in IR. Fatty acid synthase protein, encoded by *Fasn*, is an enzyme that participates in the synthesis of long‐chain saturated fatty acids. Interestingly, it has been reported that a luteolin‐enriched artichoke leaf extract reduced *Fasn* gene expression and triglyceride levels in vivo by modulating lipogenesis and fatty acid oxidation, contributing to the amelioration of liver steatosis (Kwon et al., 2018).

Thus, we hypothesized that lupin protein isolate (LPI) exerts a beneficial effect on IR and steatohepatitis in an in vivo model through the modulation of *Fasn*, *Gys2*, and *Gsk3b* gene expression. Here, we aimed to analyze the effects of LPI on IR and steatohepatitis in vivo. We also studied how lupin protein influences the expression of genes involved in lipid and carbohydrate metabolism such as *Gys2*, *Gsk3b,* and *Fasn* in the livers of animals under a pathological IR state.

## 2 MATERIAL AND METHODS

### 2.1 Plant material and lupin protein isolate preparation

Certified *L. albus* seeds were kindly provided by Dr. Edzard van Santen (College of Agriculture, Auburn University, Alabama, USA).

The dehulled *L. albus* seeds were ground into flour and defatted with hexane in a Soxhlet apparatus for 12 hr. The protein isolates were prepared as described in Figure S1 (D’Agostina et al., 2006). At the end of the extraction process, the isolates were freeze dried for 8 hr at −50°C and 0.036 mbar using a 4.5 L freeze dryer (LABCONCO).

### 2.2 High‐fat and high‐cholesterol diet

We purchased the casein, cholesterol, vitamin, and mineral mixes from Dyets, Inc. and crude sodium cholate from Sigma‐Aldrich. We then prepared a high‐fat, high‐cholesterol experimental diet as previously reported (Magaña‐Cerino et al., 2020). The diet provided 5.1 kcal/g energy, with proteins, carbohydrates, and fat supplying 18%, 22%, and 60% of the total dietary energy, respectively. The lard used in this study consisted mainly of saturated fatty acids (37%) and monounsaturated fatty acids (45%). In addition, a proportion of cholesterol and sodium cholate was added. Table 1 contains the detailed diet ingredients and chemical composition analyses.

**TABLE 1:** Formulation and chemical composition of the experimental diets

Ingredient (g/kg)	HFD	*SD* ^a^
Casein	269.0	241.0
Starch	197.0	219.0
Sucrose	111.0	315.0
Fat	—	94.0
Lard^b^	355.0	—
Vitamin Mix	10.0^c^	d.s.^d^
Mineral Mix	36.0^c^	d.s.^d^
Cholesterol	16.0	0.201^e^
Sodium cholate	5.0	—
Total energy (kcal/kg of diet)	5,100.0	3,350.0

Proximate analysis (dry basis)	Percentage	
Crude protein (*N* × 6.25)	22.9	23.0
Crude fat	35.4	4.5
Carbohydrates	38.3	58.5
Crude Fiber	0.0	6.0
Ash	3.4	8.0

Abbreviations: HFD, high‐fat diet; *SD*, standard chow diet.

^a^ Commercial diet.

^b^ Ingredients for the HFD diet only.

^c^ HFD: the values shown are in accordance with AIN‐93 recommendations for growing rodents (Reeves et al., 1993); for the *SD*, vitamins and minerals are described separately ^d^(d.s.) according to the Laboratory Rodent Diet supplier (5001).

^e^ In contrast to the HFD, the *SD* contained 201 ppm of cholesterol.

### 2.3 Animals

Male Wistar rats, provided by the Universidad de Guadalajara bioterium, were housed in an air‐conditioned room (24 ± 2°C, 55 ± 5% humidity) with a 12 hr light/dark cycle and free access to food and water. The Universidad de Guadalajara Ethics Committee approved the experimental protocol (C.I./023/2014), and all experimental procedures were conducted in accordance with the International Guidelines for Care and Use of Laboratory Animals and the Mexican Official Standard 062 (NOM‐062‐ZOO‐1999).

### 2.4 IR induction and experimental procedure

The study consisted of a 5‐week IR induction period followed by a 3‐week experimental phase. In the induction period, a group of ten healthy rats (180–220 g body weight) were provided with free access to a high‐fat, high‐cholesterol diet (HFD) to induce IR, whereas a control group (Ctrl) of five rats were fed a standard chow diet (Purina LabDiet^®^ 5001). After 5 weeks, we randomly assigned the induced IR rats to an IR‐LPI (lupin‐treated) or IR (saline placebo‐treated) experimental group (five rats per group). During the 3‐week experimental phase, animals in the IR‐LPI and IR groups were maintained on the HFD and given daily doses of LPI (2 g/kg BW of LPI dissolved in 5 ml saline) or sterile saline (5 ml), respectively. The Ctrl group was maintained on standard chow diet.

**FIGURE 1:**
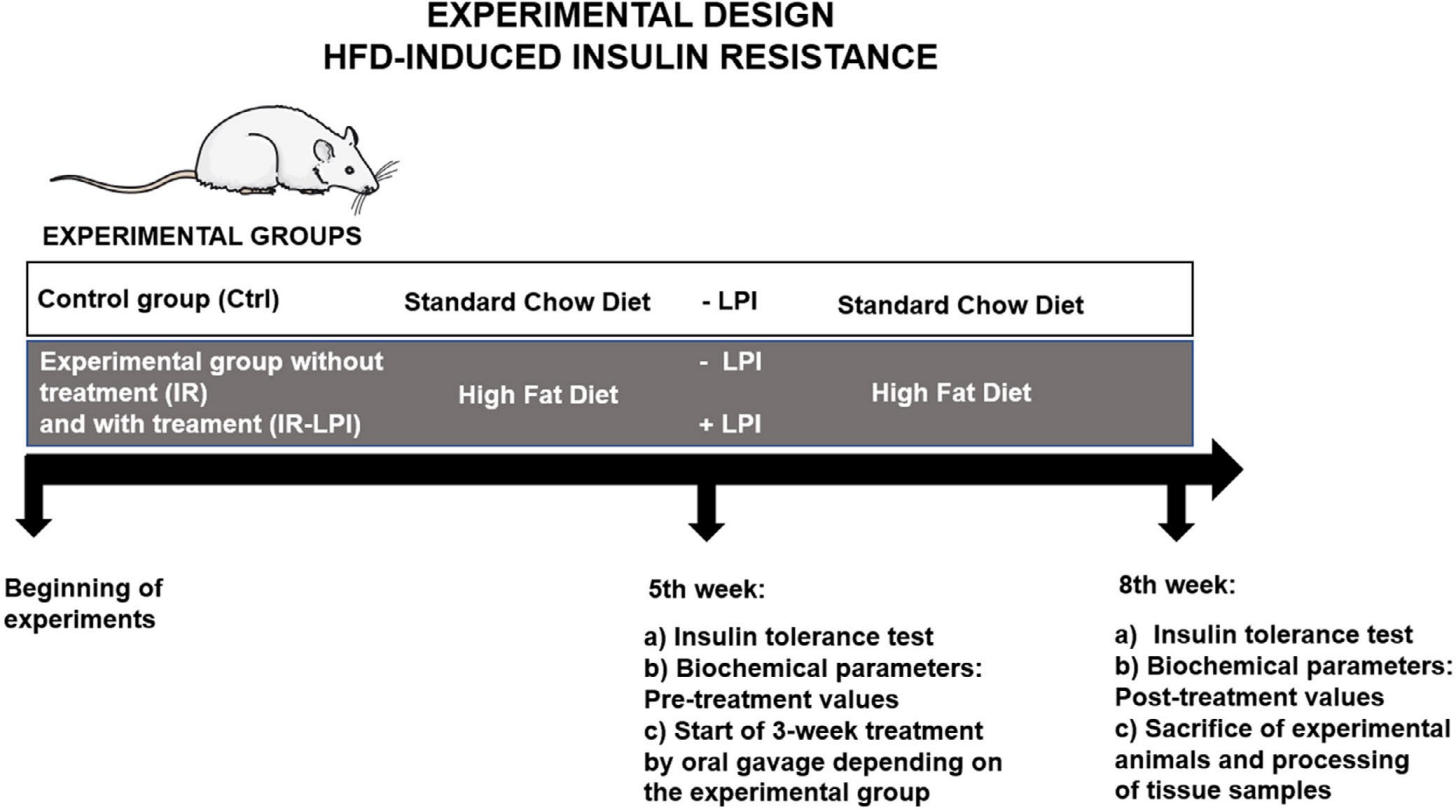
Schematic representation of the experimental design established for the study. Ctrl, control group; IR, insulin resistance group; IR‐LPI, insulin resistance group treated with LPI; LPI, lupin protein isolate

We performed an insulin tolerance test (ITT) at the start and end of the induction period as well as at the end of the experimental phase. We also determined the blood biochemical parameters at the beginning and end of the experimental phase. At the end of the treatment period, we excised and collected the livers for histological analysis and quantification of *Fasn*, *Gys2*, and *Gsk3b* gene expression (Figure 1).

### 2.5 Insulin tolerance test

The IR status was verified in the animals by performing ITT at the beginning and end of the 5‐week IR induction period as well as at the end of the treatment period. ITT was done by administering intraperitoneal insulin injections (0.5 IU/kg BW, Humulin R^®^, Eli Lilly and Company) after a 12 hr fasting period. Blood glucose concentrations were measured using a glucometer (One Touch Ultra^®^, Johnson & Johnson) at 0, 30, 60, and 90 min after insulin injection. Finally, we calculated the area under the curve (AUC) using the glucose values and determined the IR status using the Ctrl group as a reference.

### 2.6 Blood sample collection

The animals were fasted for 12 hr and anesthetized via intraperitoneal administration of Zoletil^®^ 50 (80 mg/kg BW; tiletamine‐zolazepam; Virbac). Blood samples were withdrawn from the retro‐orbital plexus. Serum was collected from the centrifuged blood samples (1,300 × *g* for 10 min, 4°C) and stored at −70°C until analysis.

### 2.7 Determination of biochemical parameters

Serum glucose, triglyceride, total cholesterol, LDL‐c, HDL‐c, and alanine aminotransferase levels (ALT) were determined using a semi‐automatic spectrophotometer (BTS‐350) and commercial reagents (BioSystems). The very low‐density lipoprotein cholesterol (VLDL‐c) concentrations were calculated using the following formula: [VLDL‐c] = ([TG]/5).

### 2.8 RNA extraction, reverse transcription, and quantification of *Fasn*, *Gys2*, and *Gsk3b* gene expression

RNA was isolated from the liver tissue using the RNeasy^®^ Mini Kit (QIAGEN). The total RNA (2 μg) was reverse‐transcribed using the Transcriptor First Strand cDNA Synthesis Kit (Roche) following the manufacturer's instructions. *Fasn*, *Gys2*, and *Gsk3b* gene expressions were quantified via real‐time PCR using the LightCycler^®^ FastStart DNA Master Plus SYBR Green I Kit (Roche) and *Rps18* as the housekeeping gene. The primer sequences for all evaluated genes were as follows: *Fasn* forward (F) 5′‐TCGAGACACATCGTTTGAGC‐3′, reverse (R) 5′‐CCCAGAGGGTGGTTGTTAGA‐3′; *Gys2* F 5′‐TCCGCTCTCCAGATGATTCT‐3′, R 5′‐GAAAAGCCCTGCTCAGTGTC‐3′; *Gsk3b* F 5′‐AGACCAATAACGCCGCTTCT‐3′, R 5′‐TGACCAGTGTTGCTGAGTGG‐3′; and *Rps18* F 5′‐CATGTGGTGTTGAGGAAAGCAG‐3′, R 5′‐GGGATCTTGTATTGTCGTGGGT‐3′. The reaction conditions were as follows: 95°C for 10 min and 45 cycles at 95°C for 10 s, 60°C for 10 s, and 72°C for 7 s for *Gys2*; 95°C for 10 min and 45 cycles at 95°C for 10 s, 61°C for 10 s, and 72°C for 5 s for *Gsk3b*; 95°C for 10 min and 45 cycles at 95°C for 10 s, 60°C for 10 s, and 72°C for 7 s for *Fasn*; 95°C for 10 min and 45 cycles at 95°C for 10 s, 61°C for 10 s, and 72°C for 6 s for *Rps18*. In the negative controls, sterile water was used instead of the cDNA. All amplification reactions were performed in triplicates using a 2.0 LightCycler^®^ instrument (Roche). We normalized the target gene Ct values with the *Rps18* Ct values and calculated the relative *Fasn*, *Gys2*, and *Gsk3b* gene expressions using the 2^−ΔΔCt^ method (Livak & Schmittgen, 2001). Single product amplification for each gene was confirmed via melting curve analysis.

### 2.9 Liver histological assessment

At the end of the 3‐week experimental period, the animals were anesthetized, and their livers were excised by laparotomy. Next, we fixed tissue fragments in 4% paraformaldehyde (1× phosphate‐buffered saline) and embedded them in paraffin. A certified pathologist evaluated the 4‐μm thick paraffin‐embedded liver tissue sections separately stained with hematoxylin and eosin (H&E) and Masson's trichrome. We also semi‐quantitatively determined liver glycogen changes by staining tissue sections with periodic acid‐Schiff (PAS)‐diastase. In this staining procedure, the tissue sections were treated with diastase before application of the PAS stain. The use of a PAS‐diastase stain allowed us to differentiate glycogen from other cellular carbohydrates. A pathologist evaluated the PAS and PAS‐diastase slides wherein the loss of cytoplasmic staining after diastase treatment indicated the presence of glycogen. Representative images were acquired from each experimental group using a Motic BA410 trinocular light microscope coupled to a Moticam CMOS 5 MP digital camera and documented using Motic Plus 2.0.

### 2.10 Statistical analysis

Results are presented as the mean ± standard error of the mean. We established which biochemical parameters significantly differed between pre‐ and post‐treatment, using the dependent *t*‐test. The significance of the inter‐group effects in *Fasn*, *Gys2*, and *Gsk3b* gene expression and AUC was established using ANOVA and Bonferroni post hoc test. We used IBM SPSS Statistics software for Windows (version 20.0; NY, USA) for data analysis and considered *p* < .05 as significant.

## 3 RESULTS

### 3.1 Biochemical parameters

Table 2 shows the results of biochemical parameter tests. At the end of the IR induction period (pretreatment), both the IR (saline placebo) and IR‐LPI (lupin‐treated) groups had higher serum glucose levels (138.6 ± 12.0 and 162.6 ± 13.3 mg/dl, respectively) than the Ctrl group (58.3 ± 18.8 mg/dl). Similarly, the HFD resulted in higher blood lipid levels in the induced IR groups (IR and IR‐LPI). However, chronic administration of LPI to the induced IR animals reduced blood glucose levels by 33% (pre‐ vs. post‐treatment), whereas the IR group with no administration of LPI had an increase of 78%. Furthermore, LPI improved the blood lipid profiles of the rats. Although not statistically significant, triglyceride levels decreased by 34% (52 ± 8.1 and 34.2 ± 11.4 mg/dl, pre‐ and post‐treatment, respectively) after the administration of LPI. Moreover, we observed a significant decrease in total cholesterol (37%) in the animals treated with LPI. Accordingly, the concentration of LDL‐c decreased after LPI administration (39%, 153.9 ± 18.6 vs. 94.1 ± 9.4 mg/dl, *p* < .05). In contrast, VLDL‐c levels were significantly increased in the IR group, whereas a decrease, although not significant, was observed in the IR‐LPI group. Finally, HDL‐c levels showed an increase of 35%, although not statistically significant (Table 2).

**TABLE 2:** Comparison of pre‐ and post‐treatment biochemical parameters among study groups

	Ctrl		IR		IR‐LPI	
	Pre‐treatment^a^	Post‐treatment	Pre‐treatment^b^	Post‐treatment	Pre‐treatment^b^	Post‐treatment
Glucose (mg/dl)	58.3 ± 18.8	67.5 ± 14.8	**138.6 ± 12.0**	246.2 ± 17.9*	**162.6 ± 13.3**	108.8 ± 13.3*
Triglycerides (mg/dl)	24.8 ± 7.4	20.8 ± 2.2	**24.6 ± 6.8**	72.4 ± 10.9*	52.0 ± 8.1	34.2 ± 11.4
Total cholesterol (mg/dl)	48.3 ± 10.4	51.3 ± 5.9	201.8 ± 41.6	162.0 ± 4.3	**223.4 ± 26.9**	140.4 ± 10.9*
VLDL‐c (mg/dl)	3.5 ± 0.8	4.2 ± 0.4	**4.3 ± 0.9**	14.5 ± 2.1*	10.4 ± 1.6	6.8 ± 2.3
LDL‐c (mg/dl)	5.2 ± 2.4	4.3 ± 2.5	170.6 ± 43.3	127.2 ± 8.5	**153.9 ± 18.6**	94.1 ± 9.4*
HDL‐c (mg/dl)	40.0 ± 8.5	44.7 ± 3.1	26.0 ± 2.5	27.6 ± 3.2	20.8 ± 3.6	28.0 ± 7.2
ALT (U/L)	62.5 ± 12.6	50.7 ± 3.0	**136.0 ± 18.4**	199.8 ± 15.3*	205.4 ± 27.9	190.0 ± 20.4

Note

Values represent the mean ± *SEM*.

ALT, alanine aminotransferase; Ctrl, control group; HDL‐c, high‐density lipoprotein cholesterol; IR, insulin resistance; LDL‐c, low‐density lipoprotein cholesterol; VLDL‐c, very low‐density lipoprotein cholesterol.

^*^Student *t*‐test for dependent samples: Statistically significant changes pre‐ vs. post‐treatment (bold text, *p* < .05).

^a^Pre‐treatment values without IR induction.

^b^Pre‐treatment values after 5 weeks of experimental IR induction.

### 3.2 Evaluation of insulin sensitivity

**FIGURE 2:**
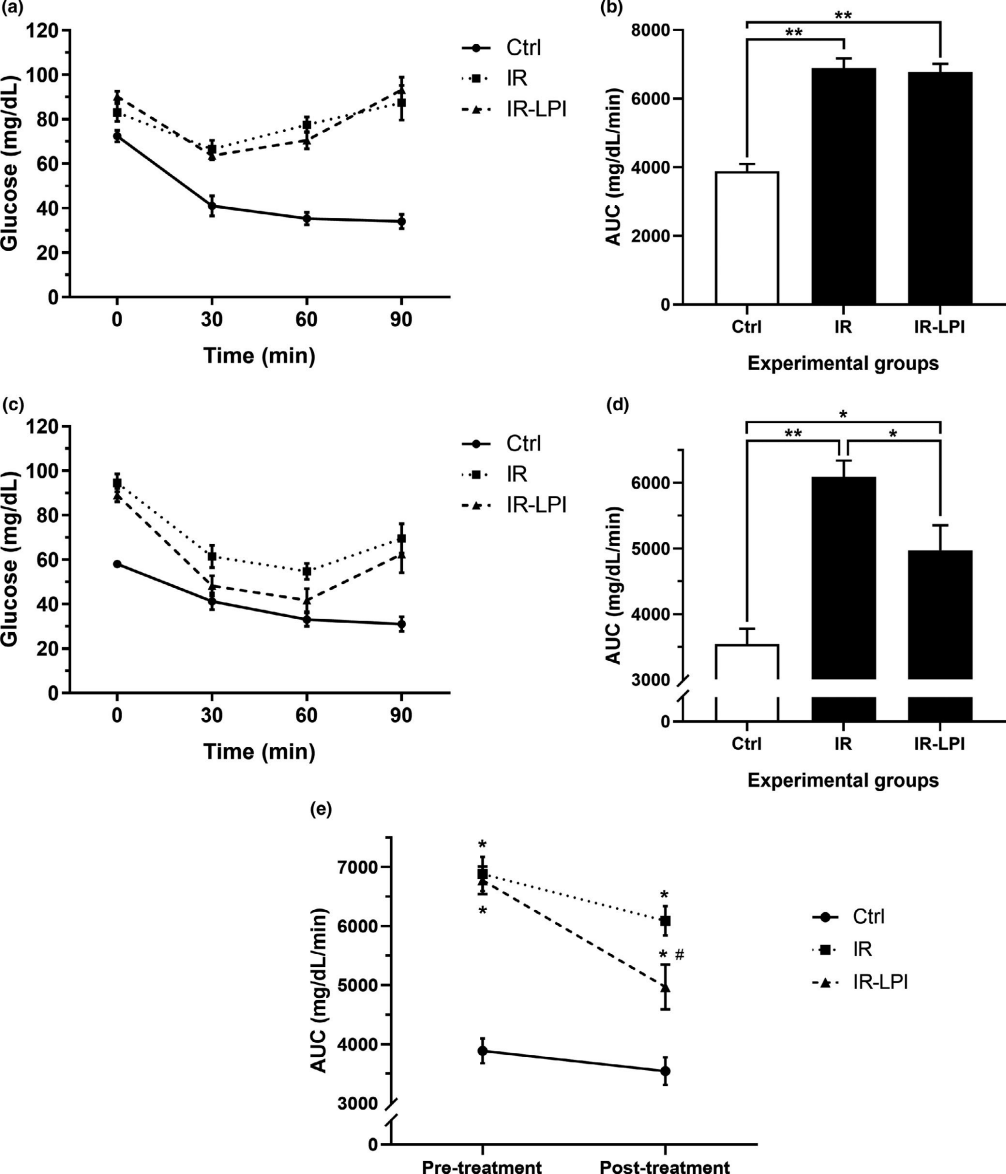
Insulin tolerance test (ITT) comparison among the experimental groups. (a) Comparison of the pre‐treatment glucose levels at basal, 30, 60, and 90 min in all groups. (b) Area under the curve (AUC) values of serum glucose concentration obtained during the pre‐treatment ITT in all study groups. (c) Comparison of post‐treatment glucose levels at basal, 30, 60, and 90 min in all groups. (d) AUC values of the serum glucose concentration obtained during the post‐treatment ITT in all study groups. (e) Summary of pre‐ vs. post‐treatment changes in the AUC values of all groups. The values are shown as mean ± *SEM*. Mean AUC values were compared using ANOVA with Bonferroni post hoc test. Panels a–d: *p <.05, **p <.01. Panel e: *p <.05 compared with control group; ^#^ *p* <.05 comparing pre‐ vs. post‐treatment AUC values using Student's *t*‐test for dependent samples. Ctrl, control group; IR, insulin resistance group; IR‐LPI, insulin resistance group treated with LPI; LPI, lupin protein isolate

Insulin sensitivity was evaluated by comparing the AUCs of the ITTs of each study group (Figure 2). As expected, the animals fed the HFD for 5 weeks developed IR. It was observed that the pre‐treatment AUC values of IR and IR‐LPI were higher (*p* < .05) than those of the control group (Figure 2b), which confirmed the development of IR in our experimental model. In contrast, the comparison of post‐treatment AUCs between the IR‐LPI and IR groups showed that the administration of LPI caused an increase in insulin sensitivity as compared to the IR group (Figure 2d). In Figure 2e, the changes in the values corresponding to the AUC (pre‐ vs. post‐treatment) of each study group are shown. The change in the pre‐ vs. post‐treatment AUC values of the IR‐LPI group was statistically significant (6,777 ± 232 vs. 4,971 ± 379, *p* < .05).

### 3.3 Gene expression

**FIGURE 3:**
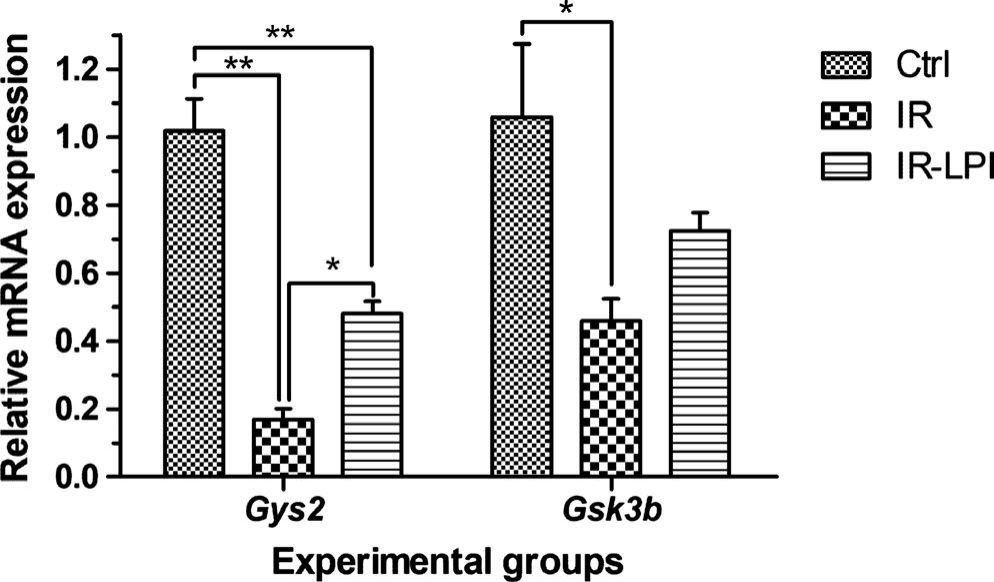
Relative mRNA expression levels of the *Gys2* and *Gsk3b* genes. The bars represent the mean ± *SEM*. We performed ANOVA and Bonferroni post hoc tests. *p <.05, **p <.01. Ctrl, control group; IR, insulin resistance group; IR‐LPI, insulin resistance group treated with LPI; LPI, lupin protein isolate

The effects of LPI on *Gys2* and *Gsk3b* gene expression are shown in Figure 3. The induction of IR caused a decrease in *Gys2* expression (83.3%, *p* < .01). Interestingly, we observed that LPI administration partially reestablished *Gys2* expression (2.8‐fold increase) in the IR‐LPI group (*p* < .05) as compared to the IR group. Our results showed that IR induction also decreased *Gsk3b* gene expression (56.6%) compared with the Ctrl group (*p* < .05). This IR‐mediated reduction in expression was attenuated in the IR‐LPI group. This shows an augmented, although not significant, *Gsk3b* gene expression after LPI treatment in comparison with the IR group (Figure 3).

**FIGURE 4:**
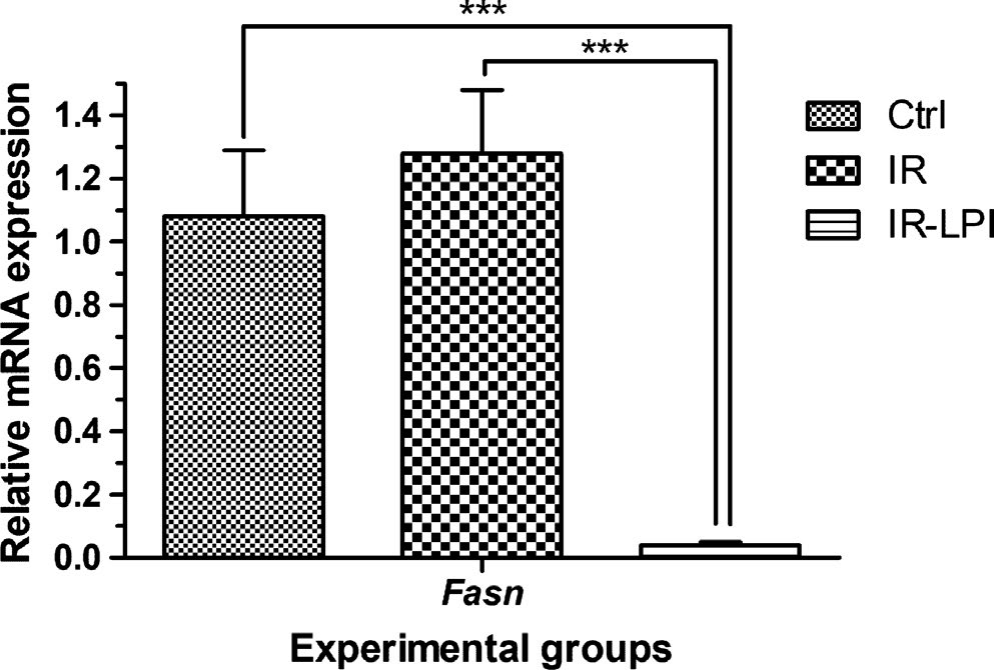
Relative mRNA expression levels of the *Fasn* gene. The values represent the mean ± *SEM*. Statistical analysis was performed via ANOVA and Bonferroni post hoc tests. ***p <.001. Ctrl, control group; IR, insulin resistance group; IR‐LPI, insulin resistance group treated with LPI; LPI, lupin protein isolate

Furthermore, expression of the *Fasn* gene in the IR group was slightly increased. However, the expression of *Fasn* in the IR‐LPI group was significantly reduced as compared to both the IR and Ctrl experimental groups (Figure 4, *p* < .001).

### 3.4 Histological analysis

**FIGURE 5:**
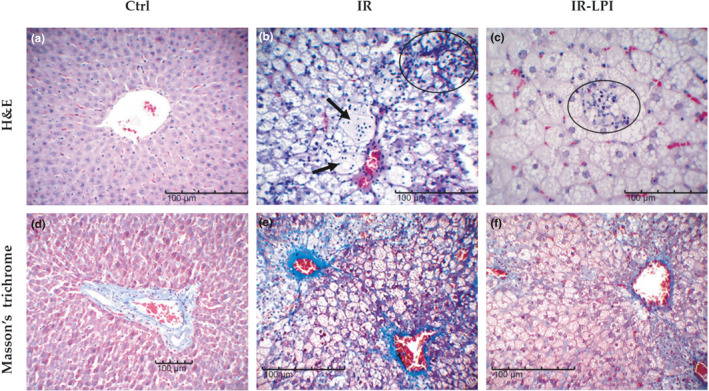
Representative photomicrographs of the histological changes observed in the experimental groups. The upper panels (a–c) show images from tissues stained with hematoxylin and eosin (H&E), and lower panels (d–f) are representative images of tissues stained with Masson's trichrome. (a) and (d) represent normal histology of the liver tissue (Ctrl group). In panel b, the figure shows the presence of inflammatory cell infiltration, a representative finding of NASH in the IR group (black circle); the arrows indicate ballooning of hepatocytes. Panel c shows a lower lymphocyte infiltration in the IR‐LPI group in comparison with the IR group (black circles). Panels e and f show the presence of fibrosis in both IR‐induced groups. Ctrl, control group; IR, insulin resistance group; IR‐LPI, insulin resistance group treated with LPI; LPI, lupin protein isolate

In the histological analysis of the liver sections, we found that our experimental model reproduced histopathological changes compatible with NASH in the induced IR groups (Figure 5). All IR animals not treated with LPI (IR group, Figure 5b) presented grade III steatohepatitis with necroinflammatory activity, based on the histopathological criteria stipulated by Kleiner et al. (2005). Interestingly, we observed decreased lobular inflammation in the IR‐LPI group (Figure 5c). Notably, only two out of five LPI‐treated animals had grade III steatohepatitis, whereas the other three experienced amelioration of the disease (exhibiting grade I or II steatohepatitis).

**FIGURE 6:**
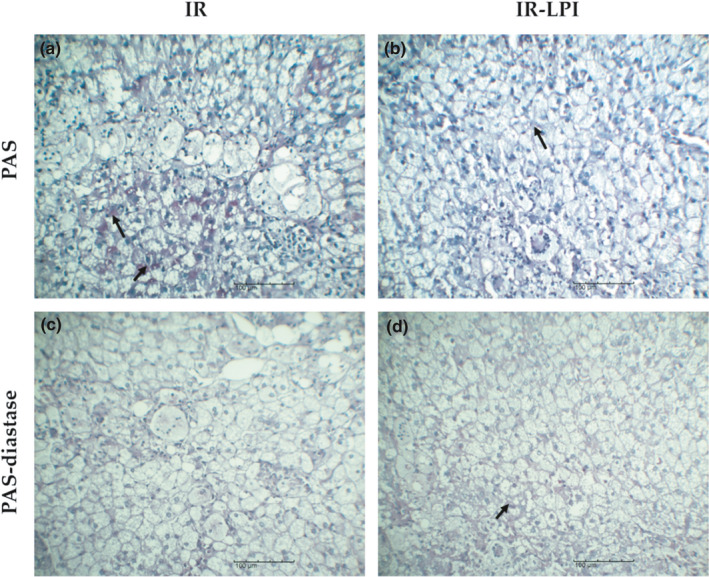
Histological comparison of glycogen content in the IR and IR‐LPI groups. Representative photomicrographs from the tissues stained with PAS and PAS‐diastase are shown. In the IR group, loss of staining in the cytoplasmic glycogen deposits (arrows) of hepatocytes after diastase treatment was observed (a and c). In contrast, panels b and d show an intercellular PAS positivity in tissues from the IR‐LPI group (arrows). IR, insulin resistance group; IR‐LPI, insulin resistance group treated with LPI; LPI, lupin protein isolate

Expression of the glycogen‐related *Gys2* gene led us to test for glycogen in liver tissue by PAS staining, wherein PAS‐positive areas exhibit a purple/magenta color. We used a PAS‐diastase staining procedure as diastase digestion causes depolymerization of glycogen into smaller sugar units with a loss of PAS positivity, allowing for the differentiation of glycogen from other cellular carbohydrates. We observed PAS positivity in the cytoplasmic area of periportal hepatocytes in the IR group (Figure 6a) and confirmed that the positivity corresponded to glycogen deposits with the diastase digestion test (Figure 6c). Furthermore, we found that the intensity of PAS‐positive areas diminished after PAS‐diastase staining in both groups (Figure 6c,d) and that the PAS‐positive areas coincided with the largest fibrosis areas. Interestingly, we were unable to detect cytoplasmic PAS positivity in liver tissues from the IR‐LPI group. However, PAS positivity was located intercellularly between hepatocytes (Figure 6b). In this case, PAS staining remained even after diastase treatment (Figure 6d).

## 4 DISCUSSION

It is widely accepted that IR is associated with metabolic disorders such as hyperglycemia and hypertriglyceridemia and is essential in the development of T2D. Thus, improving a patient's insulin sensitivity is crucial for preventing the progression of IR to NCD. This progression is associated with hyperglycemia and lipid alterations, such as increased LDL‐c and triglyceride levels as well as reduced HDL‐c concentrations (Marotta et al., 2010).

To evaluate the effects of nutraceuticals and functional foods on metabolic pathways related to IR, in vivo and in vitro models were employed. In this study, we used a high‐fat, high‐cholesterol diet to induce IR. As expected, we observed that the induced IR animals exhibited higher glucose, triglyceride, total cholesterol, and LDL‐c serum levels. The observed variations in the IR rats before the treatment could be explained by individual metabolic responses to in vivo IR induction.

Several studies have confirmed the presence of a hypoglycemic effect on humans from plant proteins, including lupin proteins (Baldeón et al., 2012; Bertoglio et al., 2011; Bouchoucha et al., 2016; Dove et al., 2011; Fornasini et al., 2012). This effect is attributed mainly to γ‐conglutin (Cγ; Bertoglio et al., 2011; Lovati et al., 2012; Magni et al., 2004; Vargas‐Guerrero et al., 2014); therefore, scientific interest has been focused on the biological effects of this protein. Cγ is a protein fraction contained in lupin seeds together with α, β, and δ‐conglutins (Duranti et al., 2008). Magni et al. (2004) demonstrated that Cγ reduced acute serum glucose levels in vivo. After publication of this study, several groups have attempted to discover the mechanisms behind the regulation of glucose homeostasis by Cγ.

Recent reports have described the effects of lupin extracts on IR. Zambrana et al. (2018) described the release of insulin from isolated islets in Goto‐Kakizaki rats after administration of lupin hydroethanolic extracts. In addition, Lima‐Cabello et al. (2017) evaluated lupin seed β conglutins through ex vivo and in vitro models and found that they upregulate mRNA levels of IRS‐1 and GLUT‐4, suggesting an effect on IR and glucose uptake. Nonetheless, little is known regarding the effects of lupin protein extracts on IR. Therefore, we aimed to investigate the effect of LPI on an in vivo IR and steatohepatitis model. In this regard, the biological effects of other legumes on IR have also been previously explored. Chickpea seed flour was found to prevent development of an IR model induced by a HFD (Yang et al., 2007). Recently, Terruzzi et al. (2018) reported that lupin flour reduced HOMA‐IR values in C57BL/6 mice that were fed an IR‐inducing experimental diet.

HOMA‐IR is a mathematic model used for IR evaluation in epidemiological studies (Haffner et al., 1996; Katsuki et al., 2001). However, its applicability to experimental research has been questioned because of its weak validation for small samples (Wallace et al., 2004). ITT is a procedure used to determine insulin sensitivity in in vivo studies. This is an acceptable method for IR evaluation since it has been compared and validated against the hyperinsulinemic‐euglycemic clamp, which is the gold standard (Akinmokun et al., 1992; Bonora et al., 1989; Gelding et al., 1994; Hirst et al., 1993).

In this study, we used ITT to evaluate the effect of LPI on IR. We found a significant improvement in insulin sensitivity after LPI administration, indicating that lupin proteins exert an insulin‐sensitizing effect.

In addition to IR amelioration, an attenuated postprandial hyperglycemia was reported in a clinical study wherein oral administration of lupin kernel flour or soybean proteins was found to exert an acute glucose‐reducing effect evaluated by the oral glucose tolerance test (OGTT) in T2D patients. The authors concluded that these results were probably due to similarities in protein content between these legumes (Dove et al., 2011). Furthermore, another study has reported that rats fed with a high‐fat diet supplemented with raw chickpea seeds exhibited reduced serum glucose levels as compared with a nonsupplemented group under measurement with OGTT and ITT (Yang et al., 2007). In accordance with these findings, our results showed that LPI treatment improved IR from experimental induction by a high‐fat diet.

Insulin modulates liver glycogen synthesis by activating glycogen synthase enzyme. The *Gys2* gene encodes liver glycogen synthase, an enzyme responsible for directing synthesis of glycogen, one of the primary sources of stored energy in the body. In contrast, *Gsk3b* encodes a serine‐threonine kinase with negative regulatory activity on glycogen synthesis. Thus, an increase in liver glycogen synthesis is associated with improved glucose tolerance (Ros et al., 2010). Since treatment with LPI has exhibited improvements in insulin sensitivity, we evaluated the gene expressions of *Gys2* and *Gsk3b*.

Here, we found that LPI treatment increased *Gys2* and *Gsk3b* gene expression in the liver. Similar results were also found in the gene expression and protein levels in diabetic mice treated with *Bauhinia holophylla* extract, a plant belonging to the Fabaceae family (Camaforte et al., 2019). Furthermore, our results showed that LPI induced a recovery of insulin sensitivity and a subsequent decrease in hyperglycemia, a finding that may have involved the activation of hepatic glycogenesis. Therefore, we decided to analyze the glycogen content in liver tissues from the experimental groups. Unexpectedly, we did not observe differences in the liver PAS positivity of the IR and IR‐LPI groups. This may be attributed to the development of prominent fibrosis when inducing IR with a high‐fat diet, hindering the histological evaluation and ability to differentiate glycogen from other extracellular matrix components. To better understand the role of LPI in glycogen metabolism, future studies should consider quantifying liver glycogen levels and assessing the protein levels of GSK3β and GYS2.

We cannot rule out higher glucose uptake by other insulin‐sensitive tissues such as skeletal muscle and adipose tissue as an explanation for our observations (Petersen et al., 2007; Tachibana et al., 2014; Terruzzi et al., 2011). Based on our data, we can hypothesize a possible increase in insulin sensitivity in the adipose tissue of the LPI‐treated animals. Studies on chickpea and soybean supplementation have shown that they prevent the development of large and dysfunctional adipocytes associated with IR, supporting our hypothesis (Clark et al., 2018). Nonetheless, additional studies are needed to establish whether lupin protein exerts similar effects.

Metabolic changes associated with IR also correlate with NAFLD and its progression to NASH (Ota et al., 2007). In the present study, we also evaluated the histological changes caused by a high‐fat diet and the effect of LPI administration on liver tissue. Using the NASH staging scoring system (Takahashi & Fukusato, 2014), an amelioration of the degree of steatohepatitis in the IR‐LPI group was observed. These findings were evidenced by decreased lobular inflammation and the presence of mild necroinflammatory activity. Previous studies have described that some lupin protein fractions decrease the mRNA levels of pro‐inflammatory genes, including IFN‐γ, TNF‐α, and NF‐κB (Lima‐Cabello et al., 2017). Fontanari et al. (2012) evaluated the weight of the liver from hamsters fed with a hypercholesterolemic diet and whole lupin seeds. Their results showed that the animals fed in parallel with lupin seeds exhibited lower liver weights compared with the control group. Although we did not perform this evaluation, biochemical analysis revealed that LPI administration attenuated the increase in ALT levels by 7.4%. Interestingly, a similar effect was observed in an IR model induced by high sucrose consumption and treated with Cγ (González‐Santiago et al., 2017).

The relationship between IR and dyslipidemia is widely known (Franch‐Nadal et al., 2018; Garg, 1996). We observed a decrease in serum triglyceride levels in the IR‐LPI group compared with the IR group. These findings correlated with changes in *Fasn* gene expression, showing strikingly reduced mRNA levels in the IR‐LPI group compared with the IR and Ctrl groups. FASN, the enzyme encoded by the *Fasn* gene, participates in the synthesis of palmitate in the lipogenesis pathway. Furthermore, both hypertriglyceridemia and steatosis can be reduced by modulating lipogenesis and fatty acid oxidation (Kwon et al., 2018). Other studies have also shown that rats fed a western diet and treated with lupin proteins exhibited lower triglyceride and VLDL levels (Fontanari et al., 2012; Spielmann et al., 2007). In accordance with our results, Spielmann et al. (2007) also found lower mRNA levels of *Fasn* and *Srebp‐1c* genes as well as lower liver triglyceride content in animals treated with lupins. In addition, it has been reported that HFDs are not only associated with NAFLD development but have also been found to induce IR (Liu et al., 2016). Therefore, the insulin‐sensitizing effect observed in LPI‐treated animals and reduced *Fasn* levels might be associated with the attenuation of liver damage.

Finally, our data contribute to the understanding of the biological effects exerted by lupin proteins in a state of IR. To the best of our knowledge, this is the first study to provide evidence that LPI could be useful in the treatment of established pathological states, such as IR and steatohepatitis. Moreover, our results add to the body of evidence indicating the presence of a beneficial effect of lupins on insulin sensitivity, as suggested by some clinical studies. Likewise, there is a need for further basic and clinical research aimed at clarifying whether lupin‐based therapies are beneficial in different clinical conditions such as obesity, dyslipidemia, IR, and diabetes.

In conclusion, our results show that LPI exerts insulin‐sensitizing and hypoglycemic effects on IR‐induced rats. Moreover, administration of lupin proteins promoted a hypolipidemic effect by decreasing serum lipid levels, involving a marked reduction in the mRNA levels of the lipogenic *Fasn* gene in induced IR animals. Thus, we provide evidence that lupin proteins might be useful in restoring insulin sensitivity and attenuating the histopathological changes induced by IR. Further molecular and metabolic characterizations of the liver and other insulin‐sensitive tissues represent an exciting research area to better understand the effects of lupin proteins and their potential use in the management of metabolic disorders.

## CONFLICT OF INTEREST

The authors declare no conflict of interest.

## AUTHOR CONTRIBUTION

Carmen Magdalena Gurrola‐Díaz made substantial contributions to conception and design; Irma Catalina Soto‐Luna participated in the acquisition, analysis, and interpretation of data; Irma Catalina Soto‐Luna and Carmen Magdalena Gurrola‐Díaz drafted the manuscript; Pedro Macedonio García‐López, Belinda Vargas‐Guerrero, Tereso Jovany Guzmán, and José Alfredo Domínguez‐Rosales participated in methodology, visualization, and revision of the manuscript for important intellectual content; Carmen Magdalena Gurrola‐Díaz gave final approval of the version to be published.

## ETHICAL APPROVAL

The animal study was approved by the Universidad de Guadalajara Ethics Committee (approval number: C.I./023/2014).

## Supporting information

Fig S1
