# Supplementary figures and images for: Lupin protein isolate improves insulin sensitivity and steatohepatitis in vivo and modulates the expression of the Fasn, Gys2, and Gsk3b genes
Source: Food Sci Nutr. 2021 Mar 8;9(5):2549–60. doi: 10.1002/fsn3.2206 (PMC8116848; doi:10.1002/fsn3.2206)

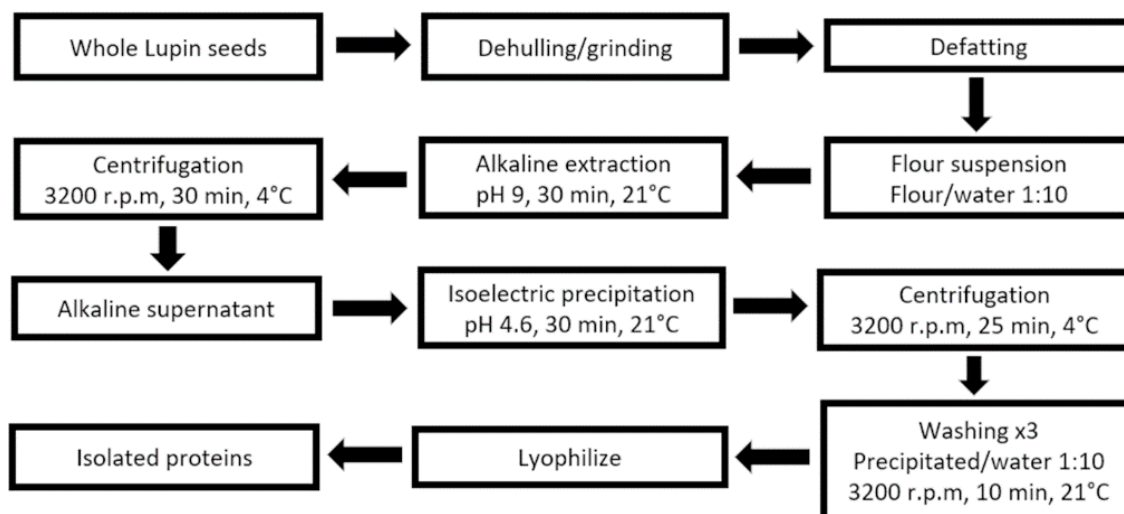

**Figure S1.** Flow chart of sequential extraction procedure for lupin protein isolate.

Supplement: Supplementary file 1 — Fig S1 [file FSN3-9-2549-s001.pdf]
